# Light-Controlled Direction of Distributed Feedback Laser Emission by Photo-Mobile Polymer Films

**DOI:** 10.3390/nano12172890

**Published:** 2022-08-23

**Authors:** Daniele Eugenio Lucchetta, Andrea Di Donato, Oriano Francescangeli, Gautam Singh, Riccardo Castagna

**Affiliations:** 1 Dip. SIMAU, Università Politecnica delle Marche, Via Brecce Bianche, 60131 Ancona, Italy; 2 Dip. DII, Università Politecnica delle Marche, Via Brecce Bianche, 60131 Ancona, Italy; 3 Department of Applied Physics, Amity Institute of Applied Sciences, Amity University, Uttar Pradesh, Noida 201313, India; 4 URT-CNR, Università di Camerino (UNICAM), Polo di Chimica, Via Sant’Agostino, 1, 62032 Camerino, Italy; 5 CNR, Institute of Heritage Science, Via Madonna del Piano, 10, 50019 Sesto Fiorentino, Italy

**Keywords:** Distriburted Feedback (DFB) laser, photomobile polymer films, holographic reflection gratings, free standing lasers, light-controlled laser direction

## Abstract

We report on the realization of Distributed Feedback (DFB) lasing by a high-resolution reflection grating integrated in a Photomobile Polymer (PMP) film. The grating is recorded in a recently developed holographic mixture basically containing halolakanes/acrylates and a fluorescent dye molecule (Rhodamine 6G). The PMP-mixture is placed around the grating spot and a subsequent curing/photo-polymerization process is promoted by UV-irradiation. Such a process brings to the simultaneous formation of the PMP-film and the covalent link of the PMP-film to the DFB-grating area (PMP-DFB system). The PMP-DFB allows lasing action when optically pumped with a nano-pulsed green laser source. Moreover, under a low-power light-irradiation the PMP-DFB bends inducing a spatial readdressing of the DFB-laser emission. This device is the first example of a light-controlled direction of a DFB laser emission. It could represent a novel disruptive optical technology in many fields of Science, making feasible the approach to free standing and light-controllable lasers.

## 1. Introduction

Polymer composites are widely used for fabrication of optical and photonic systems [1,2,3,4,5,6,7,8,9,10,11,12,13,14,15,16,17,18,19,20,21,22,23,24,25,26,27,28,29,30,31,32]. In particular, they are exploited for the fabrication of holographic volume phase gratings [3,4,12,15,17,18,26,33,34,35,36,37,38,39,40]. This class of holographic gratings represents an important platform for realizing plastic Distributed Feedback (DFB) lasers [41,42,43,44,45,46,47].

Recently, a novel class of polymers (photo-mobile polymers, PMPs) in which a mechanical motion can be induced and controlled by an external light [48,49,50,51,52,53,54,55,56,57,58,59,60,61,62,63,64,65,66,67,68,69] is emerging.

From the pioneering work of Angeloni et al. (1989, [70]), PMPs are mainly realized by using azobenzene-based liquid crystal polymers. Actually, the polymer chains are realized by azobenzene basic molecule with acrylate functions in 4, 4${}^{\prime }$ [48,49,71]. In this case, the resultant of the light-induced cis-trans isomerization of the azobenzene-molecules in the system brings to a macroscopic motion of the polymer film under irradiation. Another way to realize PMPs is based on the use of polymer mixtures where multi-acrylate molecules bring to cross-linked polymer films [51]. Many papers also report about gratings recorded on PMP-films as surface relief gratings [72,73,74,75,76]. Our group has recently reported on the recording of holographic volume transmission gratings in PMP-films [50,53,77]. However, as far as we know, the recording of an efficient reflection grating in a PMP film is still not realized. Till today the recording of a high efficiency reflection volume holographic grating in PMP films is still an open challenge. Indeed, at the time of writing, a photomobile structure containing a high-resolution reflection grating does not exist. The aim of our work is to fabricate an optically pumped DFB laser whose emission direction is controlled by an external (coherent or incoherent) low-power light source. To realize such an optically controlled DFB system two requirements must be satisfied: a) the recording of a dye-doped holographic volume reflection grating; b) the inclusion of the recorded grating in the PMP film. The first requirement can be easily satisfied by using a recently developed DFB laser device [46]. The second requirement is achievable due to the presence of unreacted acrylate-groups at the edge of the grating area that are still available for a further photo-polymerization step after the grating formation. In short, we realized a haloalkane-acrylate based polymer grating capable of DFB-lasing and covalently linked to a PMP film. Such a device allows the low power light-controlled readdressing of the DFB laser emission.

## 2. Materials and Methods

### 2.1. Materials

di-pentaerythritol-penta/hexa-acrylate monomer (DPHPA, refractive index n = 1.49, at 20 °C and λ = 589 nm), 1-bromo-butane (n = 1.437–1.441), 1-bromo-hexane (n = 1.448), 2,6-bornanedione (camphore-quinone, CQ), lead(IV) oxide (PbO ${}_{2}$), 1-ethenylpyrrolidin-2-one (or N-vinyl-pyrrolidinone, NVP), phenyl-bis (2,4,6-trimethylbenzoyl)phosphineoxide (Irg 819), 4-aminophenol (4-AP) are purchased from Merck; 9-[2-(ethoxycarbonyl)phenyl]-N-ethyl-6-(ethylamino)-2,7-dimethyl-3H-xanthen-3-iminium chloride (Rhodamine 6G) by Kodak.

### 2.2. Methods

#### Holographic Halolakanes/Acrylate-Based Mixture Preparation

The procedure involves the following four steps: (1) 69%*w*/*w* of DPHPA, 20%*w*/*w* of 1-bromo-hexane and 10% *w*/*w* of 1-bromo-butane are blended together at room temperature; (2) after that, 1% *w*/*w* of photo-initiator CQ is added; (3) the system is left under stirring for one hour until a transparent low-viscous syrup is obtained; (4) finally, $10^{-3}$ M of Rhodamine 6G is added and the mixture is left under stirring for another hour. A mixture’s droplet is placed in a sandwich made by two microscope glass slides separated by 50 μ m thick mylar spacers and irradiated in the holographic set-up at λ = 457.9 nm.

### 2.3. PMP Mixture Preparation

In a small bottle, 0.25 mmol PbO ${}_{2}$ and 1 mmol 4-AP are placed; 5 mmol of NVP are furthermore added. The reaction is left in aerobic conditions, in darkness, under magnetic stirring for seven days. After that, the precipitate is carefully removed. Separately, 1 mmol of DPHPA is blended with 0.14 mmol phenyl-bis(2,4,6-trimethylbenzoyl) phosphine-oxide and then is left, in darkness, for 3 h under magnetic stirring. The final PMP-mixture is obtained by mixing the components together. The system is left for 7 days under magnetic stirring in darkness and aerobic conditions, at room temperature.

### 2.4. Hologram Recording and Optical Characterization Set-Up

The holographic recording set-up working in reflection geometry is reported in the Supporting Information Section (Figure S1). To record the grating we used a droplet of the DFB haloalkane/acrylate based-mixture placed in the center of a sandwich cell. The entire process is monitored by a spectrometer connected to a PC (see Experimental Section and Figure S1 in Supporting Information). Two continuum s-polarized laser beams at $\lambda_{w}$ = 457.9 nm are used to write a 1D holographic grating in our sandwich-like cell. The irradiated area has a diameter d = 5 mm, and the writing power is P = 150 mW per beam. When the 1D interference pattern impinges on the holographic photo-polymerizable material, it promotes the polymerization of the illuminated areas and the phase separation between the different components of the starting mixture takes place [26,78]. The resulting high-resolution grating is a periodic distribution of inert-compound-rich domains corresponding to the dark regions of the interference pattern, and solid polymer-rich domains corresponding to the illuminated areas of the impinging light pattern. During the grating recording process, a low power incoherent white light passes through the sample and is collected by an optical fiber spectrometer. Transmission spectra are acquired and stored on a personal computer for further analyses. In this way, it is possible to detect real-time changes of the optical properties of the recorded periodic structures. In particular, the growth of the reflection peak and the amplitude related to the diffraction efficiency of the grating can be monitored each 100 ms.

### 2.5. Sample Preparation

PMP mixture is inserted by capillarity in the sandwich containing the recorded grating. In a few minutes, the PMP-mixture surrounds the spot containing the grating. The system is furthermore polymerized by irradiation with a UV-A lamp (λ = 365 nm; P = 0.5 W) for 20 min to form the PMP-DFB film [79]. After that the cell is placed in the DFB-laser set-up.

### 2.6. Pumping Set-Up (See Figure S2 in the Supporting Information Section)

Concerning the lasing effect, a frequency-doubled Nd:YAG laser impinging at 45${}^{\circ }$ with respect to the direction of the grating wave-vector $\vec{K}$ having a pulse intensity in the energy range between 77 mJ and 120 mJ (τ = 4 ns, λ = 532 nm) is used as excitation source (See Figure S2 in the Supporting Information section). The light emitted from the grating along the $\vec{K}$ direction is collected and focused by a short-focal length lens into the entrance slit of a spectro-photometer. The grating vector is equal to $\left|\vec{K}\right|=\frac{2\pi }{\Lambda }$ in which Λ is the grating pitch. In its reflection band the grating works as a DFB structure that provides a gain sufficient to observe light amplification along the optical path [46]. If the light emitted by the excited dye molecules is resonantly amplified the lasing action is feasible. When lasing occurs, the onset of a narrow peak in the emission spectrum is expected at a wavelength corresponding to one of the two edges of the Photonic Band-Gap (PBG) [45]. The Rhodamine 6G dye is chosen because at the wavelength λ = 457.9 nm its absorption band does not significantly overlap the absorption band of the used photo-initiators [45].

## 3. Results and Discussion

**Figure 1:**
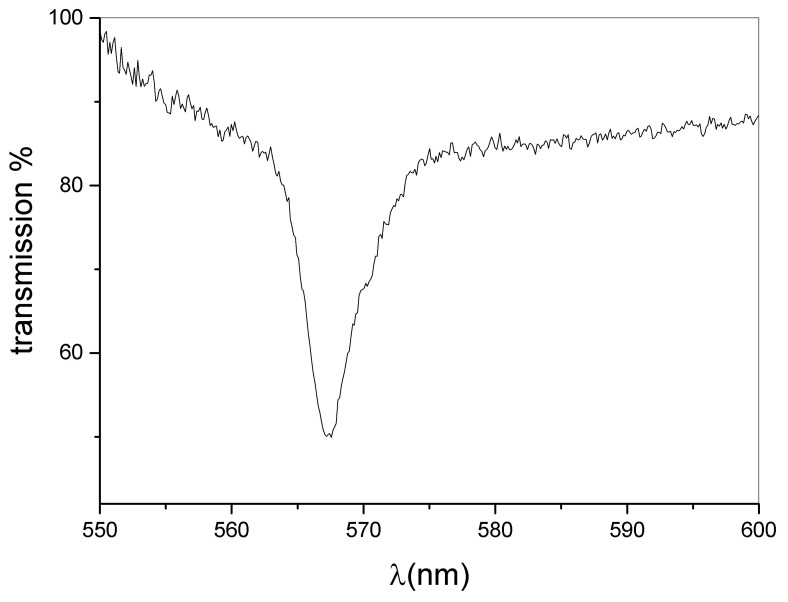
Typical normalized transmission spectrum showing the normalized reflection peak after the end of the recording process.

**Figure 2:**
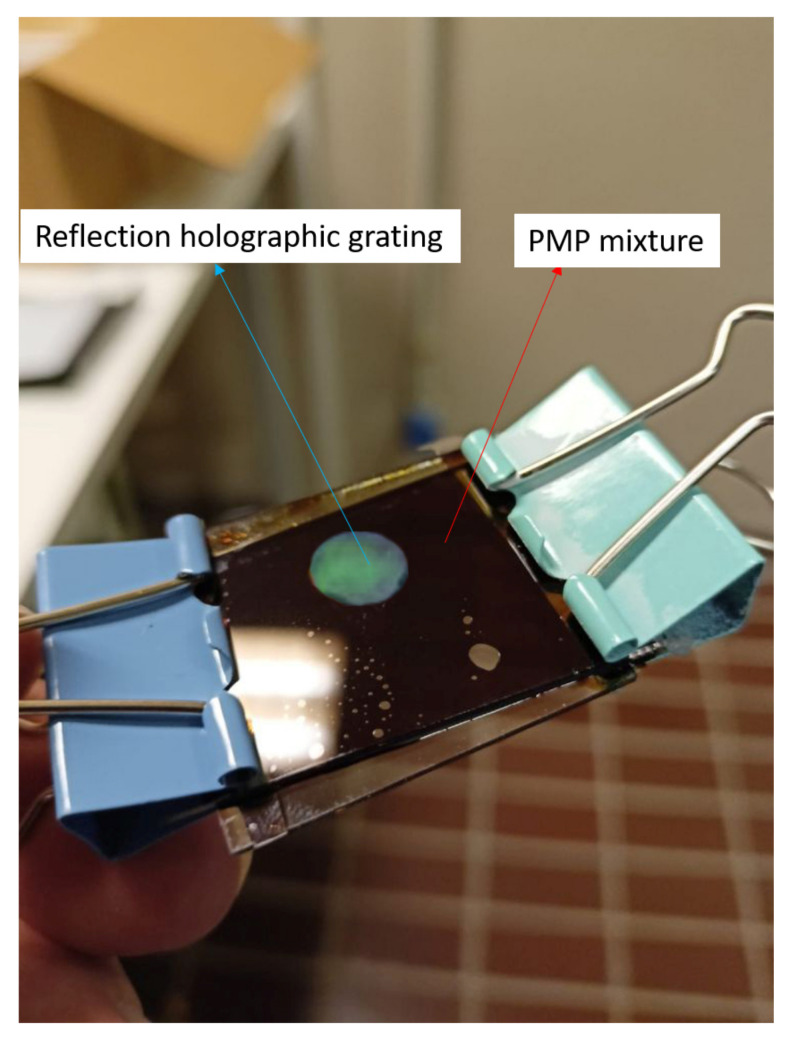
The high resolution reflection grating (the green spot) embedded into the photomobile polymer (brown part).

**Figure 3:**
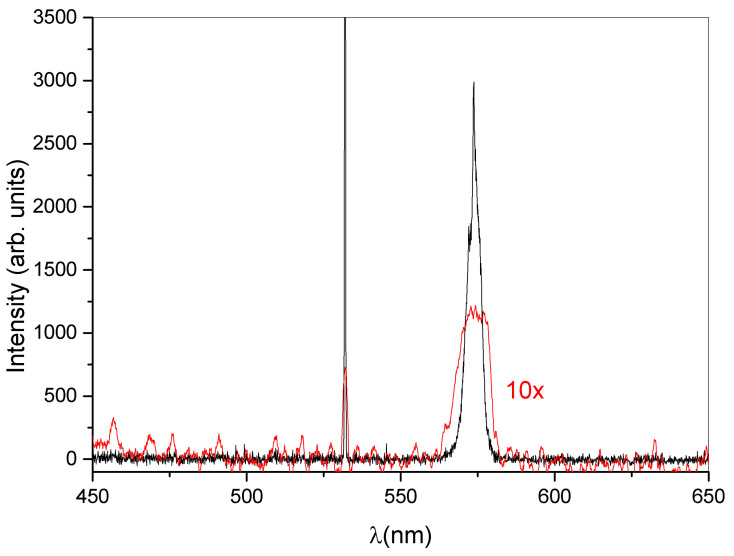
Lasing from the DFB structure under pulsed pumping at λ = 532 nm, pumping energy = 120 mJ. The narrow peak on the left side is due to the pump beam. The red line represents a 10× magnification of the typical emission spectrum of our mixture when pumped at low power values.

**Figure 4:**
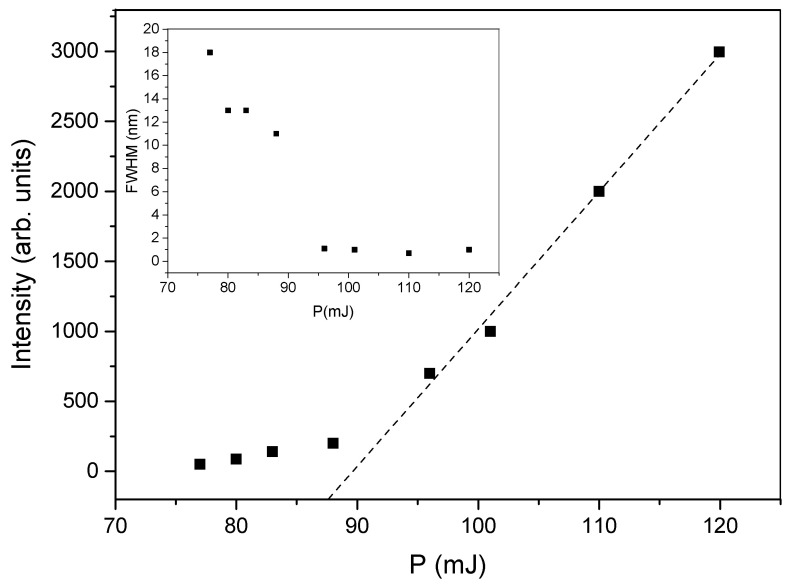
Typical peak intensity as function of the pumping energy P. The inset shows the behavior of the full width at half maximum (FWHM) with increasing the pumping energy. The dashed line is a linear regression that helps the eye in finding the laser threshold.

**Figure 5:**
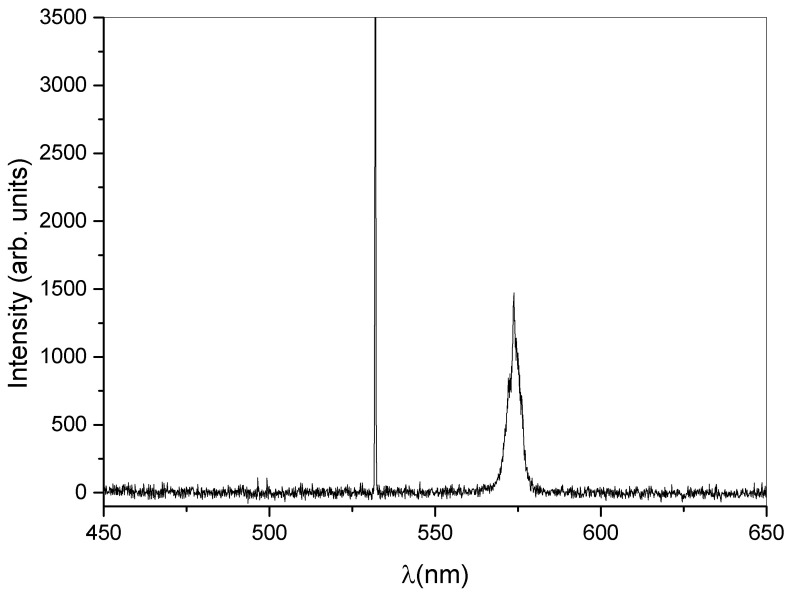
Lasing from the DFB structure after the bending of the structure under pulsed pumping at λ = 532 nm, Pumping energy = 120 mJ. The narrow peak on the left side is due to the pump beam.

The central idea of the present work resides in the realization of a DFB laser based on a dye-doped holographic reflection grating embedded in a PMP film. The direct recording of a reflection high resolution hologram in a PMP film, at present, is not possible. This is due to the nature of the PMP material and to the geometrical configuration used. A large amount of the impinging radiation is indeed reflected by the microscope slide glasses forming the sandwich cell (see Section 2). The remaining part of the impinging light is transmitted through the sample. This fraction of light is partially absorbed and induces a symmetric flow of the photo-phobic part of the mixture towards the center of the cell, making impossible the formation of the polymer walls of the grating. On the contrary, we succeeded in writing high resolution transmission gratings, directly in PMP films [50,52,53,77]. Our approach overcomes these limits, by introducing a recently developed mixture suitable for DFB-laser fabrication [46]. In this work, we used a haloalkane/acryate-based mixture that is easy and fast to prepare and shows high transparency once polymerized. Other holographic mixtures based on HPLCs [45] or TPMTGE/Acrylate [39,80] can be also suitable for the same purpose, but require a much longer preparation time. A typical experimental result concerning the recording process is shown in Figure 1. The figure reports a transmission spectrum acquired at the end of the grating formation. The measurement is normalized to the value of sample transmission spectrum before the laser irradiation. The depth of the reflection peak gives a measure of the grating diffraction efficiency which reaches values near to 30% when the sample is properly positioned. The final peak position (nm) reflects the modifications of the grating pitch Λ, due to the polymer shrinkage [81,82]. Once chosen the writing wavelength (λ = 457.9 nm for our mixture), the final peak position depends on the angle between the two s-polarized polymerizing beams. By varying this angle, it is possible to change the grating pitch and therefore the reflected wavelength. In our experimental conditions, the emission band of the dye-doped mixture containing the Rhodamine 6G ranges from 560 to 580 nm. For this reason, we decided to record a reflection peak centered at ≈570 nm. This explains the typical iridescence of the DFB structure clearly visible in Figure 2 as a green circular spot recorded in the center of the sample. After the recording process, the grating is linked to the PMP film through a further photo-polymerization process at λ = 365 nm and P = 0.5 W, see Sample preparation section and Figure S3 Supporting Information. Acting this way, at the same time we have the formation of the PMP-film and its link to the grating border. The PMP-film containing the grating is now ready to be peeled-off from the glass substrate and bent in a free standing configuration by using an external low-power light source [50,51,52,53,79] (see Figure S2 in the Supporting Information). At this stage the PMP-DFB film can be irradiated by an external pulsed laser that induces lasing at the edge of the photonic band-gap [46]. Figure 3 shows the laser emission under pulsed irradiation at λ = 532 nm. The pumping energy is P = 120 mJ for the free standing unaltered PMP-DFB film. As reference a 10X magnification of the emission band of the mixture below the laser threshold is shown in red. The corresponding measured laser threshold is reported in Figure 4 together with the behavior of the FWHM. The dashed linear regression gives a measure of the lasing threshold that is ≈88 mJ. In the inset of the same figure, it is reported the behaviour of the FWHM as function of the pumping energy. A reduction from tens of nanometer to ≈1 nm is observed above the threshold value of 88 mJ. To bend the PMP-DFB film an external continuous incoherent UV light at (λ = 356 nm; P = 0.5 W) is used. The PMP-DFB bends and the pulsed pumping process at λ = 532 nm is repeated. In this situation, the DFB laser is displaced from the collecting optical fiber detector, resulting in a lower lasing intensity (for comparison purposes, the result is shown in Figure 5 with the same scale of Figure 3). When the incoherent light is switched-off, the grating comes back to the initial position and the DFB laser measured intensity becomes maximum again.

## 4. Conclusions

Fabrication and characterization of a DFB laser integrated in a recently developed photo-mobile polymer film are reported. The PMP-DFB film easily bends under the action of an external incoherent low power light. This bending allows the light-induced displacement of the DFB-laser emission. This novel technology opens new perspectives in the fields of optical communications, integrated optics and opto-electronics.

## Data Availability

Not applicable.

## Supplementary Materials

The following supporting information can be downloaded at: https://www.mdpi.com/article/10.3390/nano12172890/s1, Figure S1: Recording Set-up; Figure S2: Lasing Set-up; Figure S3: Chemicals
