# Supplementary material for: Light-Controlled Direction of Distributed Feedback Laser Emission by Photo-Mobile Polymer Films
Source: Nanomaterials (Basel). 2022 Aug 23;12(17):2890. doi: 10.3390/nano12172890 (PMC9458089; doi:10.3390/nano12172890)
Supplement: Supplementary file 1 [file nanomaterials-12-02890-s001.zip › nanomaterials-1887053-supplementary.pdf]

Supplementary Materials

Light-Controlled Direction of Distributed Feedback Laser Emission by Photo-Mobile Polymer Films

Daniele Eugenio Lucchetta [1,*] , Andrea Di Donato [2] , Oriano Francescangeli [1], Gautam Singh [3] and Riccardo Castagna [4,5,*]

1 Dip. SIMAU, Università Politecnica delle Marche, Via Brecce Bianche, 60131 Ancona, Italy
2 Dip. DII, Università Politecnica delle Marche, Via Brecce Bianche, 60131 Ancona, Italy
3 Department of Applied Physics, Amity Institute of Applied Sciences, Amity University, Uttar Pradesh, Noida 201313, India
4 URT-CNR, Università di Camerino (UNICAM), Polo di Chimica, Via Sant'Agostino, 1, 62032 Camerino, Italy
5 CNR, Institute of Heritage Science, Via Madonna del Piano, 10, 50019 Sesto Fiorentino, Italy
* Correspondence: d.e.lucchetta@staff.univpm.it (D.E.L.); riccardo.castagna@cnr.it (R.C.).

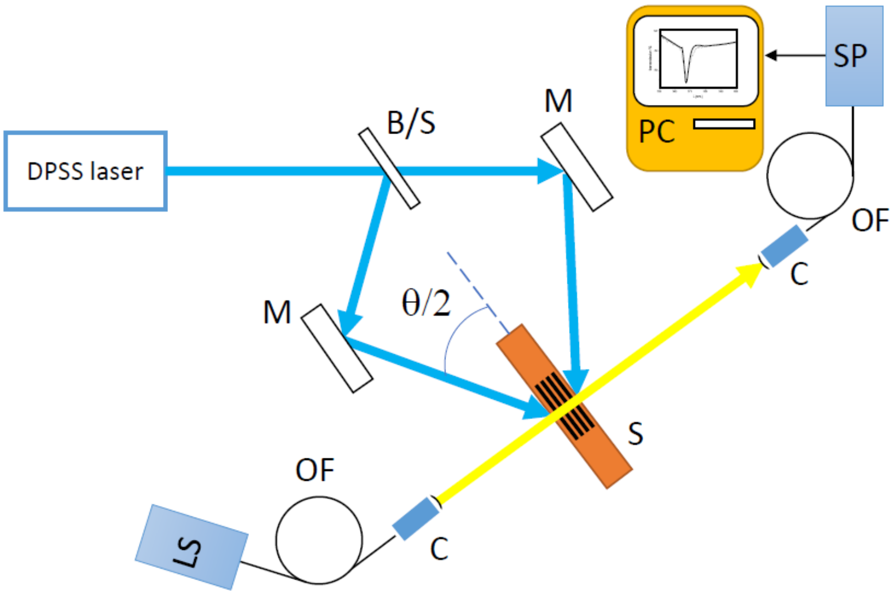

Figure S1. Schematic representation of the writing setup for the high resolution reflection gratings. B/S: beam splitter; M: mirror; LS: light source; OF: optical fiber; C: collimator; S: sample (holographic reflection grating); SP: spectrometer; PC: Personal Computer.

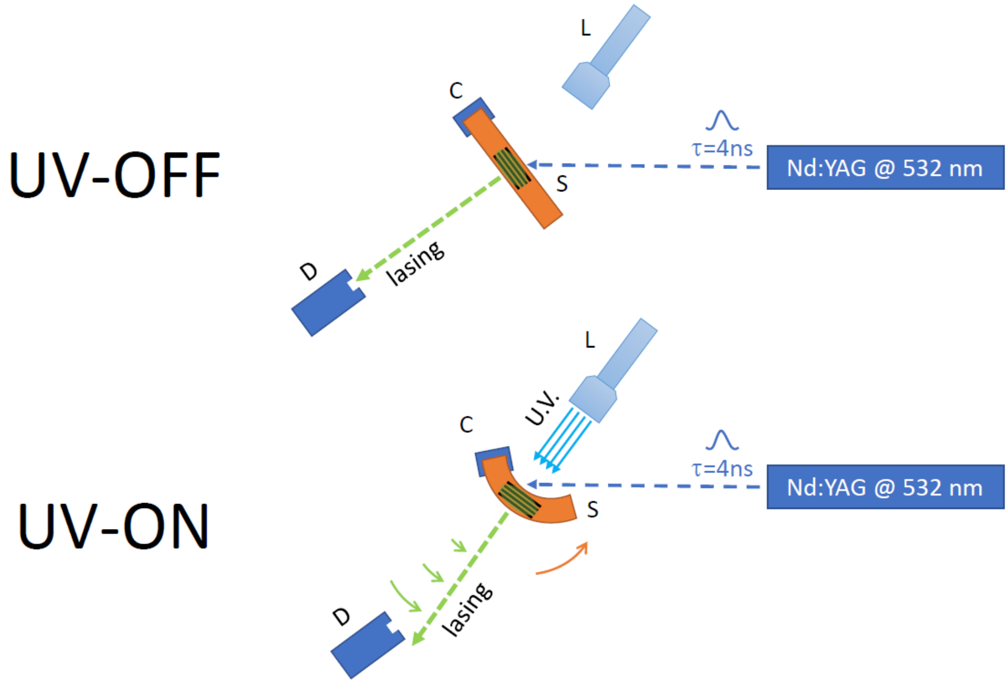

Figure S2. Schematic representation of the pumping setup and lasing action. S: sample (holographic reflection grating); C:PMP film clamping; D: photodetector; L= U.V. Lamp.

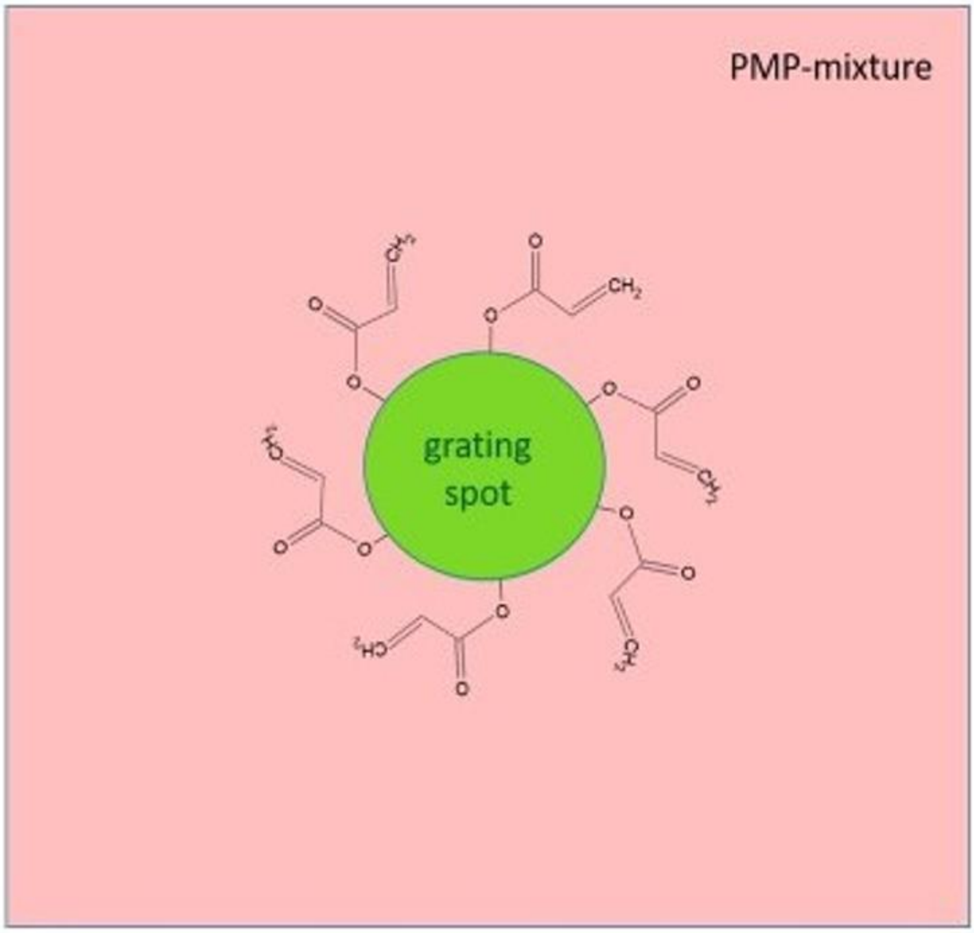

Figure S3. Schematic representation of the free acrylate functions surrounding the spot area.
